# Exploring the nature of science through courage and purpose: a case study of Nikolai Vavilov and plant biodiversity

**DOI:** 10.1186/s40064-016-2795-z

**Published:** 2016-07-22

**Authors:** Joel I. Cohen, Igor G. Loskutov

**Affiliations:** Montgomery County Public Schools, Rockville, MD USA; Graduate School USA, Washington DC, USA; Audubon Naturalist Society, Chevy Chase, MD USA; Department of Genetic Resources of Oat, Barley, Rye, N. I. Vavilov Institute of Plant Genetic Resources (VIR), 44, Bolshaya Morskaya Str., St-Petersburg, Russia 190000

## Abstract

**Introduction:**

Historical biographies facilitate teaching the ‘nature of science’. This case study focuses on how Nikolai Vavilov’s unrelenting sense of purpose, courage, and charismatic personality was maintained during violent revolutionary change in Russia.

**Case description:**

The rediscovery of Gregor Mendel’s laws of inheritance provided Vavilov with a scientific foundation for crop improvement, this foundation was later bolstered by Vavilov’s personal drive to conserve plant biodiversity. As he advanced theories and pragmatic approaches for genetic improvement and conservation of plants, political leaders in Russian came to reject Mendel’s principles and eventually Vavilov’s work.

**Discussion and evaluation:**

This rejection occurred because Joseph Stalin was desperate for a quick remedy to the famine and suffering from forced collective agriculture. Vavilov’s work continued, modernizing Russian crop research while inspiring other scientists to save seeds stored in the world’s first gene bank. Three themes illustrating the nature of science help examine Vavilov’s life: explaining natural phenomena, uncompromising human endeavor, and revising scientific knowledge.

**Conclusions:**

The case study concludes with four questions to stimulate student inquiry and self-guided research. They also deepen student understanding of Vavilov’s personal sacrifices to ensure use and conservation of plant biodiversity.

## Background

This case study examines the pioneering work in crop plant improvement and conservation undertaken by Nikolai Ivanovich Vavilov and how he succeeded in doing so against overwhelming odds. To accomplish this, Vavilov relied on his unrelenting purpose and internal drive, combined with an infectious personality that motivated and inspired others to stake their very lives on such a man. Incorporating a “nature of science” approach to Vavilov’s life and work offers a unique way to examine his accomplishments and his visionary leadership.

The three themes explaining the nature of science in this case study were identified and explained by the Next Generation Science Standards (NGSS Lead States 2013, McComas 2015). These include: Theme 1: Science as a human endeavor, Theme 2: Science models, laws and theories explain natural phenomena, and Theme 3: Scientific knowledge is open to revision in light of new evidence.

Using biography to amplify relevance and meaning of scientific discovery in the classroom is possible (i.e., Clough 2016). However, such materials are rare and not suited for immediate classroom use. When they are available, they often cannot be used in deference to ensuring full coverage of a prescribed curriculum. Thus, it is easier to omit teaching of the perilous events scientists often face, and their courage to do so, in order to advance the very theories students must learn to graduate. A second topic of importance, now recognized by the NGSS and of growing instructional importance, includes biodiversity, although educators wishing to elaborate or extend upon textbook-based lessons find few options (Navaro-Perez and Tidball 2012). One brief example is offered for conservation methods (Hawtin and Cherfas 2003).

The Next Generation Science Standards (NGSS 2013) influence what is taught and assessed in science curricula, and its focus on biodiversity is included in Core Idea LS2, Ecosystems: Interactions, Energy, and Dynamics. Using case studies allows topics such as biodiversity to be taught in relation to the nature of science and by emphasizing the human elements of science (McComas 2015). This way, human dimensions invoking “individual struggle, creativity, and adventure” are incorporated into science teaching (Eldridge 2009). This case study fulfills this need specifically for secondary and undergraduate education.

The case study is also directed towards students and academicians, for as noted, Vavilov, “has largely passed under the radar in terms of wider public and scientific appreciation. Not only have his scientific contributions failed to be given the wider acknowledgement they so readily deserve, but his premature death was shameful” (Ling 2015). This case study, by being classroom ready, can advance this overdue acknowledgement by introducing Vavilov to the coming generations of students.

### Theme 1: Science models, laws and theories explain natural phenomena

#### Explaining natural phenomena: part 1—Vavilov’s ‘Centers of Origin’

The year was 1924 and Nikolai Vavilov was ready to usher in a concept new to science. In that year, Vavilov reorganized the Bureau of Applied Botany to become the All-Union Institute of Plant Industry,^1^ for which he was Director, located in what is now St. Petersburg. His directorship duties multiplied, and by 1934 Vavilov had founded over 400 research institutes, with staffing requirements of 20,000 (Janick 2015).

Building upon studies, explorations, research and his travels abroad, Vavilov was now sure that scattered across the globe existed geographical “centers of origin and diversity” for our major food crops, and that these did not occur at random. With this pronouncement and its publication, he had simultaneously built on and transcended the earlier, pivotal works of Darwin (2008, 1868) and De Candolle (1914). Within these centers, lay a cauldron of genetic interplay stretching back thousands of years, sometimes intersecting with humanity and other times, left to itself.

Vavilov (1932) came to recognize that, “One of the most essential factors in understanding the process of evolution in living organisms is the geographical distribution of species and varieties at the present time and the past.” Vavilov always recognized the contributions of others, such as for Darwin, he said: “Darwin’s theory of evolution is a cornerstone, being the basic and unique theory that has been standing solidly for more than 80 years. In their professional activities, botanists, zoologists, geneticists, plant breeders and ecologists as well as plant geographers are influenced by this universal theory, and it is only due to this fact that understanding the process of evolution and the functioning of organisms becomes possible,” (Lostokov 1999, p. 17).

Vavilov went further than just explaining the diversification and evolution surrounding food crops. He used this concept as a “scope” along which he could systematically direct plant collection and exploration, especially in his centers of origin. What are now called “Vavilovian Centers of Origin” underwent four major revisions. He initially postulated three centers in 1924, then five, ending with eight in 1934, and eventually reducing to a final number of seven in 1940. With this interpretation and revision of his first work on centers, we see a man of vision, working a theory until it made sense with his own observations and with his understanding of development from diversity. Each revision represented the product of detailed analysis of all the new information coming in from his studies and travels.

As summarized by Harlan (1992), Vavilov believed that “the geographic region in which one found the greatest genetic diversity was the region of origin.” Within each of these centers lay not only the origins of our food crops, but other sources of diversity that included plant species not cultivated but related to a certain crop, and other species that were far more distant in their genetic makeup. To say this idea and recognition were new, or to say they were a breakthrough would be gross understatements. Instead, these theories were genius, coming from Vavilov’s tireless curiosity and bravery, never being afraid to question himself, always searching the corners of the globe for insight and evidence.

Reaching back in time to explain where and how a crop entered this world becomes complex all too soon, as seen when reading *Evolution of Crop Plants* (Simmonds 1976). This book presents the evolution of plants one crop at a time, including factors as the geography of the site where agriculture arose and if an indigenous civilization was present, such that “the geography of crop variation depends a lot upon the geography of human history,” (Harlan 1992).

Vavilov postulated that to find his centers, one had to look “in those few regions where primitive agriculture was still practiced, especially in the mountains, where from earliest times people have tilled the soil,” (Popovsky 1984). Vavilov concluded that these centers stretched across several mountain ranges, from which he was able to discover the original home of many food crops, something no one else had done.

Once such understandings became clear to Vavilov, they guided collecting missions for all who worked at VIR. Vavilov altered and revised his final views on the number and detail of the centers, but the systematic method it provided the collection is what made his next theory just as unique and visionary.

#### Explaining natural phenomena: part 2—loss of genetic variation

 Vavilov now understood that crop plants could also be improved by using diversity taken from non-cultivated plant species. By sampling a crop’s biodiversity, and focusing on the wild and related species, Vavilov saw their potential use for disease and pest control and for breeding tolerance to harsh growing conditions (Plucknett et al. 1987). From this, the fundamental importance of plant improvement programs was realized, as scientists could take advantage of resistant genes derived from a wild population. Corn for example, can benefit from introduction of traits from its wild and related species (Cohen and Galinat 1984).

But to improve plants in this manner meant conserving the relative and wild species most closely related to our food crops. However, what if this diversity had already started to disappear? Vavilov had seen such disappearance due to the modernization of agriculture. Here again, Vavilov recognized something decades ahead of its time, and that was “genetic erosion.” Whether caused by humans or nature, the loss of diversity meant the erosion of the genetic base of a crop plant’s diversity (Hummer 2015).

Only once this theory and the geography of the centers of origins were understood, did Vavilov consider sufficiently prepared to launch a “vigorous, worldwide plant exploration program… and for the first time a really systematic plan for genetic resource management was established (Harlan 1992, p. 49).

Even if the centers of origin do not always correlate with areas of greatest biodiversity, the areas that Vavilov identified remain important collecting areas. Many centers are explored today and still hold diversity of wild and natural relatives of domesticated crops, and thus hold promise for future investigations of crop biodiversity (Allard 1960).

But as his work, explorations, and theories were coming together an image haunted Vavilov. He realized that the very thing he and his colleagues collected could disappear just as fast. A final challenge stood in front of him. What if he and his colleagues collected all these seeds, but could not protect them? How could they ensure that the seeds they collected would survive? How could they serve as “trusted bankers” for collections across so many crops and countries?

A daring moment, once again, Vavilov faced. Foreseeing the need to counter-act genetic erosion meant that he must somehow secure long-term protection for the diversity held inside each seed deposited country by county, trip by trip, seed by seed, into a bank such as no one had seen before.

#### Explaining natural phenomena: part 3—Vavilov—a pioneer of the genebank (1924–1944)

Why speak of banks when discussing biodiversity? Today, we take banks for granted, we deposit things of value in their safety deposit drawers, we expect it to be safe and secure there, we place our money in savings accounts, and our personal valuables in their vaults. From day to day, barring financial collapse, we expect to reclaim what we deposit as we remembered it, at whatever time in the future such needs arise.

Therefore, should it be with the diversity of life. We need such a bank for seeds, as seeds contain unique combinations of genetic diversity. Preventing the loss of seeds, being the reproductive product of plants, forestalls the loss of biodiversity surrounding our food crops. What makes such a bank possible? Banks established for agricultural biodiversity are called ‘genebanks,’ meaning a repository or storage center for many forms of plant genetic material, including seed and other reproductive tissues. Just as a normal bank keeps our personal valuables safe, a genebank keeps deposits of biodiversity that may provide valuable genes for our food crops (IPGRI 2004).

There are three overriding requirements of a genebank needed to maintain seed viability: temperature, seed moisture, and the original vitality of the seed being deposited (Plucknett et al. 1987, p. 77). While these pose considerable challenges, improvements to meet these challenges have advanced as well, leading to the longer-term storage conditions of modern genebanks. Forty years after Vavilov initiated seed conservation, the introduction of modern varieties in tropical countries raised anew concerns regarding loss or displacement of local crops and seeds. Russia, the United Kingdom, and crop specific genebanks supported by the Rockefeller Foundation together provided a platform for the expansion of genebanks that began in the 1950s (Pistorius 1997). This led to the development of numerous national and international genebanks that ushered in the era of modern conservation (Hawtin and Cherfas 2003; Cohen et al. 1991; Plucknett et al. 1987).

The idea would emerge in someone’s mind, coming foremost to one who travelled in search of the rare and disappearing. It was Vavilov who took this imperative of conservation and translated it into one of the first genebanks in modern times (Janick 2015), at once providing a means for saving seeds for perpetuity. Vavilov, ever the pioneer, would never know that what followed his efforts at Leningrad would eventually lead to hundreds of other genebanks around the world.

When Vavilov reorganized the Bureau of Applied Botany, the first major genebank in the world was also established. Even though Vavilov gave the construction and operation of this center high priority, the facility was only able to store seeds at room or ambient temperatures, as cold storage refrigeration was not yet available. Thus, to keep these deposits viable, that is not to lose their ability to germinate and grow, they had to be planted out each year (Plucknett et al. 1987). The harvested seeds were returned to the seedbank for deposit.

By the 1940s, VIR scientists began experimenting on genebank operations to improve long-term storage to ensure that the deposits in the genebank, and at 40 satellite collections and breeding stations, would stay viable longer than year to year. Eventually, VIR scientists determined optimal conditions for storing seed and other planting material (Loskutov 1999). However, genebank deposits were not only to be conserved, but used. Once stored in the Leningrad genebank, Vavilov trusted that other scientists would see his deposits as a “genetic insurance policy” for future crop improvement. As such, Vavilov insisted that the seed being collected enter into evaluation, screening and crop improvement programs as soon as possible. Through these efforts, Vavilov established plant breeding^2^ programs and over 100 experimental stations across Russia, based on principles of Mendelian genetics and on the type of seed collections VIR.

Results from plant breeding take several years to see and to be confident of the advantages of the new crops. It is the genetic backbone of crop improvement and Vavilov saw that each of his research institutes was involved. It was in this manner that he hoped to avert worsening food shortages and increase farmer output. However, Joseph Stalin grew impatient with this timeframe, and labelled Vavilov a failure because results were promised only years into the future.

Just as the imposed reality of Stalinism closed in on those who had disposed of the tsar and ushered in the Russian revolution, so did Stalin close in upon those who no longer met with his expectations. This eventually included Vavilov, whose persecution seems such an incongruous and pointless attack. However, to the Russian dictator, nothing was further from the truth, even though his case against the seed collector was built on false hopes and Stalin’s growing misguided favoritism towards another Soviet scientist named Trofin Lysenko, as will be seen next.

### Theme 2: Vavilov’s contributions to science arose from human endeavor

Nikolai Vavilov seems a contemporary in theory and practice rather than someone of a century past. Yet, Vavilov’s story and sacrifices remain unknown in the world of secondary and undergraduate science education. What Vavilov accomplished came from human endeavor, put forward under some of the most arduous conditions imaginable. As presented by the NGSS (2013), scientists, “rely on human qualities such as persistence, precision, reasoning, logic, imagination and creativity, and are guided by habits of mind such as intellectual honesty, tolerance of ambiguity, skepticism and openness to new ideas.” To discuss Vavilov’s endeavors, persistence, and contemporary relevance, this theme begins with Vavilov’s efforts in conserving plant biodiversity (as it would be called today).

#### Human endeavors: Part 1—Vavilov and biodiversity

The genebank that Vavilov had conceived opened its doors in 1920s Petrograd (as the city was called then, replacing its original name of St. Petersburg, only later to become Leningrad). The All Union Institute of Plant Breeding (VIR), with Nikolai Vavilov serving as its Director, now had a bank different from all others. Its value rested not in gold or silver, but in the plant seeds stored within. By 1941, seed from more than 187,000 varieties of plants was held inside (Alexanyan and Krivchenko 1991). The dreams and prescience of Nikolai Vavilov were becoming reality. By 1975, over 230,000 deposits had been made; helping his bank to become the largest depository of seeds worldwide (Loskutov 1999).

While many have contributed valuable lessons, research and theory to the science of gene banks and crop biodiversity (i.e., Frankel and Bennett 1970), it was Vavilov who almost 100 years ago envisioned the need for and persisted to activate his vision by building the world’s first gene bank that is still operating today.

#### Human endeavors: part 2—biodiversity makes an entrance

Long after Vavilov’s genebank opened, the science of “biodiversity” was announced in September 1986 under the auspices of the National Academy of Sciences and Smithsonian Institution (NRC 1988). Since then, the term “biodiversity” continues to draw attention to the loss of species in the wild, especially those living within the great tracts of nature, places harboring the rare and endangered, and often, species not yet known to science.

When students and teachers consider biodiversity today, they often focus on animals living in wilderness, savannahs, or rainforests. When asked to think of specific species, students readily select endangered megafauna, a group of animals which captures not only their attention (Cohen 2014, 2016), but that of organizations seeking “flagship species” for conservation (Leader-Williams and Dublin 2000). What is practically unknown to students, but of great importance to Vavilov, is that the same concerns of loss and endangerment surround the preservation and conservation of our remaining food crop biodiversity. These concerns were not presented in the 1986 Academy meeting.

Why not? One reason may have been that many ecologists, environmentalists, and naturalists maintained that adoption of modern crop plants replaces rather than sustains diversity. Of those who study choices available to farmers, there is a concern that farmers will choose seed offered through institutions or public research, rather than from his or her own local seeds. If the new seeds are chosen, then it is believed that monocultures of these varieties will take over farms that otherwise would have significant amounts of plant biodiversity. If and as this occurs, the “cultivation of a small number of fast-growing varieties of crops condemn ever-larger areas of the Earth’s surface to low biodiversity,” leading to over-extraction of resources and a decline in ecosystem services provided by a more diverse habitat (Mackay 2009, p. 90).

Secondly, resources provided for agriculturally related seed conservation have had a long history of financial and curatorial support (Cohen et al. 1991), while emerging biodiversity action plans were just developing in the 1990s. Therefore, biodiversity priorities did not focus on plants having agricultural-economic value or use (Pistorius 1997). Seeds were viewed as a storehouse for agricultural diversity, not for the conservation of biodiversity.

Thirdly, the conservation of crop plant biodiversity differs from conserving organisms and habitats featured in that 1986 conference. Seeds are stored in off-site conservation collections, often far from their natural centers of origin and diversity, removed from farmers’ fields. However, this is not the only way that crop plant biodiversity can be conserved and maintained.

Subsequent work by many (Brush 2004; Nabhan 1989; Potter et al. 1993) has documented the significance of crop conservation in natural settings, mostly managed by farmers themselves. The struggle to feed the world sustainably continues. Recognizing the benefits of indigenous, local cropping systems along with the global and commercial demand for food staples guarantees a reliance on both options for famers.

**Fig. 1:**
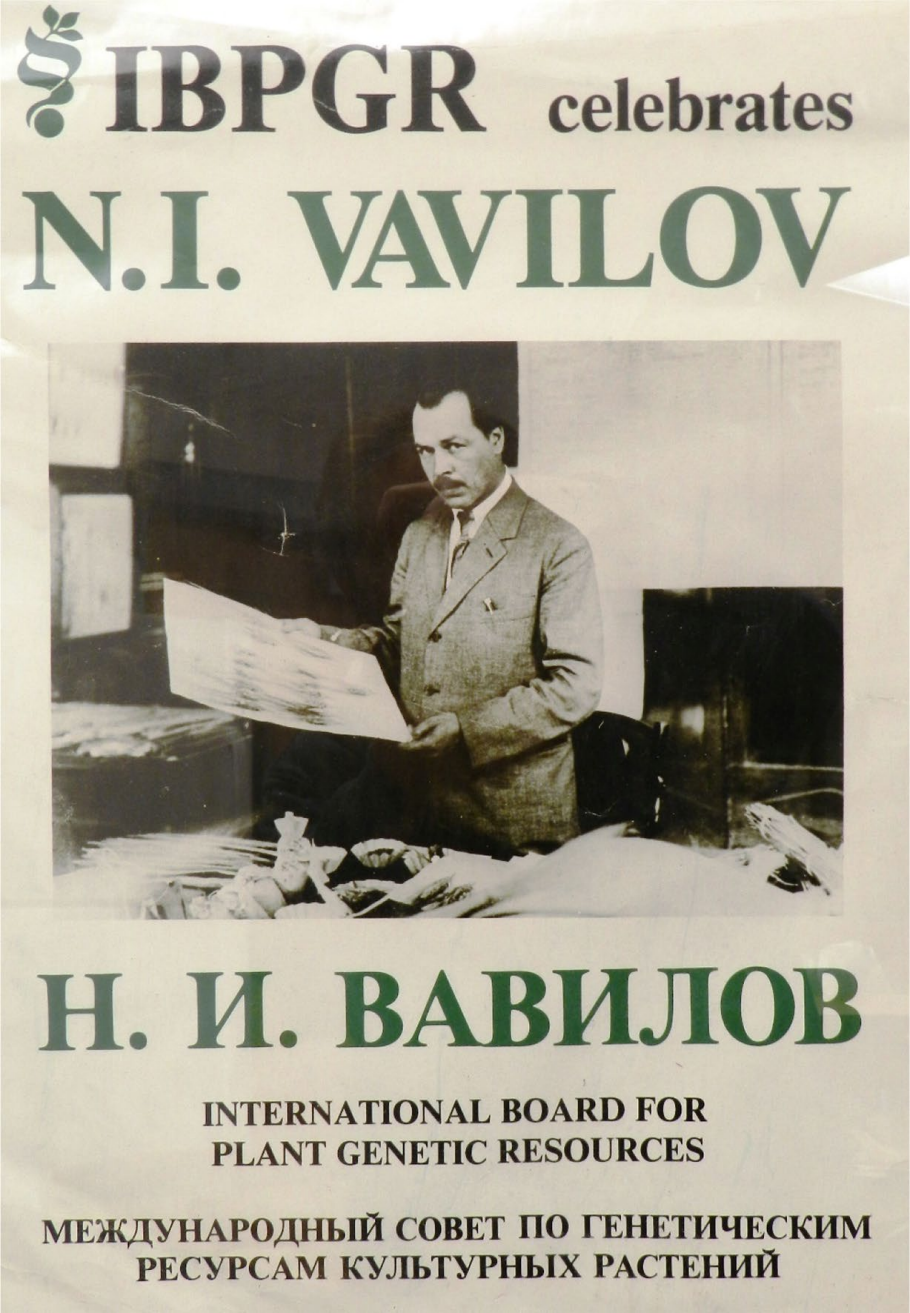
Nikolai Vavilov commemorated in a bilingual poster by the international board for plant genetic resources

Go back some 60 years before the 1986 conference, before science had distinguished on farm and off farm conservation. Here, Vavilov is seeking to resolve that difference, ensuring that conservation of local plant biodiversity had a purpose: to enhance diversity available immediately for crop improvement. This is just one legacy of Vavilov’s endeavors, as commemorated in a reprinted photograph taken in the Director’s office at Vavilov’s institute (Fig. 1).

#### Human endeavors: part 3—Vavilov’s expeditions from 1922 to 1940

Over the past several decades, plant explorers have set off on dangerous journeys to study, observe and collect food plant biodiversity. Besides Vavilov, other such adventurers are highlighted in Plant Explorers (2016) (https://www.plantexplorers.com/explorers/index.html), although these names alone do not do justice to collectors from the tropical and developing counties or to international institutes.

Perhaps what makes Vavilov unique among these collectors is not just the sizable number of plant deposits contributed, but also the sheer number of locations from which he collected. These stories are recalled in detail in *Five Continents* (Vavilov 1997). This book gives evidence of the care Vavilov took in documenting each site. His methodology, as summarized by Loskutov (1999), was to not only pay attention to the type of seeds collected, but for “… site ecology, and studies of cultivation technique, but also a geographical description of these countries and provinces and their various natural and meteorological conditions,” to name a few.

These expeditions were expedited by Vavilov’s command of several European and some Asian languages. He patiently recorded the details of each of over 100 explorations that he led across fifty countries between 1915 and 1930, covering Asia, Africa, Central and South America (Loskutov 1999; Table 1). In Vavilov’s time as Director, the total number of deposits in the bank reached 250,000. Vavilov immediately saw to it that these new sources of plant biodiversity were “thoroughly studied at different experiment stations in different geographical zones of the country,” (Loskutov 1999).

Each of Vavilov’s expeditions produced new results which were published, and garnered great attention among scientists in the West, leading to his publication of *The Geographic Origins of Cultivated Plants* in 1926. According to Popovsky (1984), “From then on, Vavilov became one of the most respected leaders of world biology.” Vavilov’s constant revision and synthesis of what he learned from these travels gradually led to visionary insights identifying specific locations where our food plants originated and diversified, as discussed in the previous theme.

#### Human endeavors: part 4—saving the seed—to the death

September 8, 1941. German troops encircled the city of Leningrad. Hitler tried to bypass it but he did not spare the city from aerial assaults and the starvation of its population. The siege lasted 900 days, ending in January 1944. By the time of the battle, the VIR genebank had accumulated seeds from 187,000 varieties of plants. Throughout the siege, the scientists at VIR sought to maintain and protect all of the crop plant biodiversity collected thus far. Because more advanced long-term storage was not yet available, the material needed periodic replanting and harvesting to ensure it remained fresh and viable. This became very risky to the scientists, and yet they managed to carry it out secretly every year.

Over one million people died because of the siege and the prolonged starvation. With no other food in sight, the VIR genebank scientists refused to eat a single seed so carefully placed inside their bank. Two of these officials, S. M. Alexanyan and V. I. Krivchenko, told of the extreme conditions they faced: “It became increasingly difficult to work in the institute. The building was unheated, as there was neither firewood nor coal. Because of unrelenting firing on the city’s center, the building’s windows were broken and had to be boarded up. The institute was “cold, damp and dark,” (Alexanyan and Krivchenko 1991). Nine of the scientists died from starvation during the siege.

By doing so, they had protected perhaps the most important contribution resulting from Vavilov’s endeavor—the most important collection of crop plant biodiversity in the world.

### Theme 3: Scientific knowledge is open to revision in light of new evidence

All scientific discoveries, theories, and data are subject to scrutiny and revision; this is a hallmark of the nature of science and the scientific method. As noted by NGSS (2013, Appendix H), “Indeed, the only consistent characteristic of scientific knowledge across the disciplines is that scientific knowledge itself is open to revision in light of new evidence.” While best done by the scientific community, it was Stalin’s impatience with the time required for Vavilov’s plant breeders that brought almost a complete turn-around in Vavilov’s standing.

From the time Vavilov was appointed Head of the Department of Applied Botany and Plant Breeding in 1820, he was in a race against time. Vavilov reached his station in Petrograd in 1921, just in time to witness that year’s catastrophic drought. Vavilov’s approach was for the long-term; he believed that there was no reason to worry about institutional time horizons, as his position was secure, and his research methods appreciated by those working under his direction. The following excerpt provides a vision of what his institute would become, if given a strong organization and adequate time:

I would like the Department to be a necessary institution, as useful to everybody as possible. I’d like to gather the varietal diversity from all over the world, bring it to order, turn the Department into the treasury of all crops and other floras, and launch the publishing of “Flora Culta”, the botanical and geographical study of all cultivated plants. The outcome is uncertain, especially considering the surrounding hunger and cold, (Loskutov 1999, p. 18).

Secondly, Vavilov would have liked nothing more than to stave off famine and starvation by improving essential food crops. This urgency was internal, fueling his desire to immediately begin the fight to end famine and poor crop yields. But, as any plant breeder can attest, such improvements are better measured in years rather than months. However, with the political rise of Trofin Lysenko, the son of a peasant, came a person promising immediate improvements based on accelerating the growth of plants by treating seeds with low temperatures and moisture. Following his work on plants in 1928, he called this process “vernalization.” Thus began the politicization of science, with Lysenko the peasant scientist on one extreme rejecting Mendelian science, and Vavilov, the progressive higly educated scientist on the other extreme, bringing modern science to his home country.

With the rise of Lysenko, Vavilov could sense a political shift coming and prepared for a purge in his institute. In the 1930s, Vavilov’s enemies took advantage of a combination of poor harvests and unclear government signals for improving agricultural production to strengthen Lysenko’s position in the Soviet State. In 1936, Lysenko had Vavilov dismissed as head of agriculture in Petrograd, giving Lysenko more freedom to profess the concept of vernalization. As time progressed however, vernalization did not contribute to increased yields that could meet such high expectations (Loskutov 1999). However, it was Vavilov, under orders from Stalin, who was eventually imprisoned in 1940 for not agreeing with Lysenko’s pronouncements, while Lysenko’s final fall from power would not occur until the 1960s, after Nikita Khrushchev served as the First Secretary of the Communist Party of the Soviet Union.

Vavilov’s commitment to gathering and using plant diversity beyond Russia’s borders was cast off by Stalin by documents and pronouncements favoring Lysenko’s seemingly immediate approach to increasing food production through vernalization. Mendelian inheritance and subsequent discoveries by Vavilov were banned in favor of a “science” that would give immediate famine relief. Vavilov and his ideas became a scientific scapegoat for the massive starvation do in part to a failing agricultural system. The scientist and his scientific research, once so proudly hailed and honored by Lenin, became contemptable to the Stalinist system. Its revision and demise led to a decline in Soviet science and agriculture that lasted for decades.

Propelled by science, Vavilov pushed on, as if all of the zeal captured in his scientific pursuits was enough to hold off the darkness that stormed around him. As noted by Zakharov (2005), “The whole life of Nikolai Ivanovich Vavilov is a remarkable example of wholehearted devotion to science, to his homeland and to humanity.” Vavilov speaks further on the centrality of his scientific pursuits by saying that, “I really have a profound faith in science, in which I find both purpose and life. And I am quite ready to give my life for the smallest thing in science,” (Pringle 2008).

### Questions to extend the case study

Educators and students can extend their studies through the following questions:

1. Using Vavilov’s ‘center of origin’ concept, create a simulation that might mitigate adverse impacts of human activity on biodiversity in and around Vavilov’s centers.
2. Compare the life of Vavilov with other scientists showing courage in defending their ideas to a hostile government, church, or society. Think here of Galileo, Darwin or Rachel Carson as examples. Compare and contrast the outcomes of each as to how they made their way in the face of such formidable opposition, often subject to prison or death.
3. Human activity is having adverse impacts on biodiversity through overpopulation, overexploitation, habitat destruction, pollution, introduction of invasive species, and climate change. How might biodiversity in our genebanks counter these adverse impacts?
4. How did Vavilov’s explorations form his conception of centers of origin and diversity?

## Conclusion

In a scientist’s life there is opportunity, and what is done with that opportunity. In Vavilov’s case, it would be hard to imagine more being done with the time and opportunities afforded him. As one example, it now takes a global effort to surpass the initial efforts by Vavilov in genebanking. These activities are carried out through a network of global, national, regional and institutional genebanks that together account for vast numbers of collected, catalogued, and conserved seeds. Without Vavilov’s systematic collecting during his explorations and his insistence on a functioning genebank, who knows how many seed deposits would have been lost forever.

What Vavilov began with one gene bank, currently totals approximately 2.7 million plant deposits in 449 institute genebanks around the world (Genesys 2016). From this seed, subsequent crop improvements are made routinely, especially in the area of disease and pest resistance, and local area environmental adaptations. In addition, molecular approaches for tapping into a gene bank’s biodiversity offer additional means for addressing the needs of future global food production for the future (McCouch 2013).

Not only was Vavilov willing to tackle the painstaking job of constructing and organizing his institute’s genebank, he was also an unfaltering mentor, motivating his staff to the highest level of performance and personal sacrifice. Finally, of lasting importance, is Vavilov’s treatise on the ‘center of origin’ of our crop plants. This is why those interested in the origin and diversity surrounding our food crops still begin their studies from Vavilov’s theories on the origin and diversity of plants.

The crop seeds, wild species and crop relatives collected by Vavilov so long ago can now be viewed as part of modern-day concerns regarding biodiversity. “Conserving biodiversity” was not an idea during Vavilov’s time. However, it certainly goes to the heart of what he achieved during his collecting trips. Vavilov sought out the theory and practice of collecting to guide the systematic deposits of seed in a genebank. Nabhan (2009) recognized the importance of these deposits, including them within “agricultural biodiversity.” also, as noted by Nabhan (2009, p. 15), “Vavilov and Harlan were among the first to articulate the concept of loss of agricultural biodiversity through … genetic erosion.”

Vavilov’s personal endeavors and unwavering consistency, equal to his theoretical contributions, was the focus of this case study. Admittedly it is somewhat artificial to categorize the life of a scientist into the NOS Matrix and its basic understandings as identified by the NGSS (2013, Appendix H). However, by using the NOS framework, it makes it possible to study Vavilov’s life in a secondary science curriculum. If this is done, studies will introduce to a new generation Vavilov’s contributions, his singular endeavors that made these possible, and the professionalism he maintained through years of demeaning persecution, his imprisonment by Stalin, and finally, his death from starvation in January 1943.

## Footnotes

1 In 1968 this institute was renamed as the N.I. Vavilov All-Russian Scientific Research Institute of Plant Industry, in honor of its 75th Anniversary. VIR will be used for this institute in the following pages of this paper.

2 Plant breeding refers to changing the traits of plants genetically, in order to produce more desired characteristics.
